# The Impact of Different Sports on Reducing Mobile Phone Addiction: A Systematic Review and Network Meta‐Analysis

**DOI:** 10.1111/adb.70087

**Published:** 2025-09-01

**Authors:** Ying Li, Jianhua Zhang, Zhaojun Luo, Dan Tao, Lizhong Wen

**Affiliations:** ^1^ College of Sports Science Jishou University Jishou Hunan China; ^2^ School of Physical Education and Arts Hunan University of Medicine Huaihua Hunan China; ^3^ Department of Physical Education Shenzhen Foreign Languages School (Group) Guangming Campus Guangzhou China

**Keywords:** mobile phone addiction, network analysis, teenagers

## Abstract

This study employed network meta‐analysis to evaluate the impact of several exercise interventions on mobile phone addiction. The aim is to determine the most effective exercise intervention measures and establish a reference for future intervention measures to improve mobile phone addiction. We systematically searched the relevant literature on the Web of Science, PubMed, Embase, Cochrane Library, China Knowledge, Wanfang and other domestic and foreign databases. We assessed the risk of bias according to the revised Cochrane Randomised Trial Bias Risk tool and performed traditional and Web‐based meta‐analyses using Review Manager 5.3 and Stata 14.0. The traditional meta‐results showed that exercise intervention was superior to the control group in improving mobile phone addiction (SMD = −1.05, 95%CI −1.62, −0.48). Network meta‐analysis results show that aerobic exercise (AE) is superior to other sports in reducing the total score of mobile phone addiction among teenagers, and the probability of aerobics becoming the best intervention for mobile phone addiction among teenagers is the highest (SUCRA = 95.6%). Exercise intervention can reduce the score of mobile phone addiction, while AE has more advantages in improving mobile phone addiction. However, due to the influence of sample size and the quality of the included literature, it is recommended that the results be further verified in the future.

## 1 Introduction

With the continuous development of information technology, mobile phones have become increasingly popular [1]. In recent years, mobile phones have become an essential part of daily life worldwide. Relevant data indicates that global mobile phone users are growing exponentially. It is estimated that by 2026 [2], international mobile phone users will reach 7.516 billion. While mobile phones bring convenience, they also bring potential risks, leading to new behavioural problems, among which is mobile phone addiction [3]. Mobile phone addiction is regarded as an impulse control disorder, defined as the inappropriate or excessive use of mobile phones, resulting in loss of control over mobile phone use [4], disruption of daily life and causing extreme emotional changes and severe physical reactions in individuals [5]. Mobile phone addiction reduces activity levels and leads to an increase in fat and a decrease in muscle mass [6]. It also lowers the sleep quality of teenagers [7], causes damage to the lens and leads to immune system dysfunction [8]. In addition, teenagers' skulls are thinner, and their brain tissue is more electrically conductive. They are more likely to absorb mobile phone radiation than adults and have a higher risk of developing brain tumours than adults [9]. In addition, mobile phone addiction can also cause anxiety in various aspects, such as self‐existence, social interaction, and academic studies. The cognitive dissonance and negative automated thinking it triggers can further exacerbate depression, leading to more suicidal thoughts among teenagers. Furthermore, mobile phone addiction is negatively correlated with the academic performance of adolescents [10]. Excessive use of mobile phones can weaken students' inhibitory control, working memory and attention, affect teaching coherence and hinder establishing a supportive, cooperative learning environment, and those at high risk also find it more challenging to improve school adaptability through self‐regulation [11]. Intervention research on mobile phone addiction primarily encompasses cognitive and exercise‐based approaches [12]. Cognitive behavioural intervention targets the identification and restructuring of maladaptive patterns related to mobile phone dependence [13], whereas exercise interventions predominantly address physiological mechanisms [14]. Current evidence suggests that exercise enhances brain function in addicts through dopaminergic modulation: physical activity stimulates pituitary dopamine secretion, which competes with addictive behaviours for central nervous system receptors, thereby generating pleasure and alleviating anxiety, stress, and negative emotions associated with internet addiction [15]. Exercise‐induced neurotrophic factors not only enhance neural plasticity and normalise cortical activity, but also improve cognitive function through sustained activation of task‐related brain regions, particularly the bilateral precuneus, thereby facilitating mental health rehabilitation [15, 16]. As far as we know, there have been systematic reviews [17, 18]. Gong et al. demonstrated the association between exercise and mobile phone addiction, but the effect of exercise on reducing mobile phone addiction among teenagers still needs further exploration [19]. Network meta‐analysis (NMA) has gained prominence in evaluating medical interventions due to its capacity to estimate the relative effectiveness and ranking of interventions, even in the absence of direct comparisons [20]. Meanwhile, NMA allows the use of indirect comparison methods to quantitatively compare different interventions for the treatment of similar conditions and thus select the best treatment regimen [21]. Therefore, the purpose of this network meta‐analysis is: [1] to quantitatively compare the impact of exercise on people addicted to mobile phones; [2] by ranking the effects of different types of sports through NMA, the most suitable sports for improving teenagers' mobile phone addiction are screened out.

## 2 Methods

This study was reported per the PRISMA NMA guidelines [22]. The review protocol was registered with the International Prospective Register of Systematic Review (PROSPERO) (CRD420251042695).

### 2.1 Search Strategy

The computer searched PubMed, Web of Science, Embase, Cochrane Library, CNKI, and other databases, and the search period was established until April 20, 2025. The search takes the form of combining subject words and free words.

### 2.2 Study Selection

The inclusion criteria for study selection were based on the PICOS methodology (participants, interventions, comparators, outcomes and study design) [22], shown in Table 1.

**TABLE 1:** Inclusion and exclusion criteria.

Category	Inclusion criteria	Exclusion criteria
Population	Teenagers who have reached the level of mobile phone addiction after assessment	Medical diagnosis of mental and psychological disorders.
Interventions	Aerobic exercise (AE), Basketball, Tai chi (TC), Table tennis (TT), Bicycle, Baduanjin, Other exercises (OE), Combined exercise (ce: two or more specific types of sports training [23])	
Comparisons	Control group (CG)	
Outcomes	Using Smartphone Addiction Scale Shortened Version (SAS‐SV), The mobile phone addiction index (MPAI)	
Study	Randomised controlled trial; published in English or Chinese	Duplicate publications; conference papers and review articles.

### 2.3 Data Extraction

Two independent reviewers extracted data, capturing the following parameters: primary author, publication year, country, and intervention duration. Quantitative outcomes were presented as arithmetic mean with accompanying standard deviation (mean ± SD). In cases where outcome measures were reported at multiple time points, data from the final assessment interval were preferentially selected for analysis.

### 2.4 Risk of Bias Assessment

The risk of bias was assessed independently by two reviewers and by a third reviewer using the tools provided by the Cochrane Collaboration [24], including sequence generation, hidden assignment, blinking, incomplete outcome data, non‐selective reporting of results, and other sources of bias. Each criterion was judged to have a low, unclear or high risk of bias.

### 2.5 Data Analysis

Meta‐analysis was performed using RevMan 5.3 software. For the scoring of adolescent mobile phone addiction, due to the significant differences in the evaluation tools used in each individual study (such as scale items, scoring criteria, etc.), the standardised mean difference (SMD) of the scores after intervention was adopted to combine the effect size. A 95% confidence interval (95% CI) represented each effect size. *I* ^*2*^ was used to determine the heterogeneity of effect indicators among different studies quantitatively. An *I* ^*2*^ > 50% or a *p* value of 0.10 or less for the Q test was interpreted as indicating substantial heterogeneity [24]. When the heterogeneity was significant, the random effects model was used; otherwise, the fixed effects model was used. Subgroup analysis was based on movement characteristics and population characteristics. The source of heterogeneity was explored through sensitivity analysis for the studies with significant heterogeneity. Sensitivity analysis was used to test whether the source of heterogeneity was due to one of the original studies.

Network meta‐analysis (NMA) was also conducted to perform a random‐effects multivariate NMA for pooled estimates within the frequentist framework [25]. The geometry of the network was summarised in a network plot, where the lines connecting nodes represented direct head‐to‐head comparisons between interventions, and the size of each node and the thickness of each line connecting the nodes were proportional to the number of studies. A network contribution graph was drawn to calculate the contribution of each direct comparison. The loop‐specific heterogeneity estimates, inconsistency model, and node‐splitting analysis were used to analyse the inconsistency between direct and indirect comparisons. The surface under the cumulative ranking curve (SUCRA) was used to rank and compare the effects of the different interventions. SUCRA values range from 0 to 100, where 100 indicates the best treatment with no uncertainty, and 0 indicates the worst treatment without uncertainty [26]. Moreover, a network funnel plot was generated to check for publication bias.

## 3 Results

### 3.1 Study Selection

**FIGURE 1:**
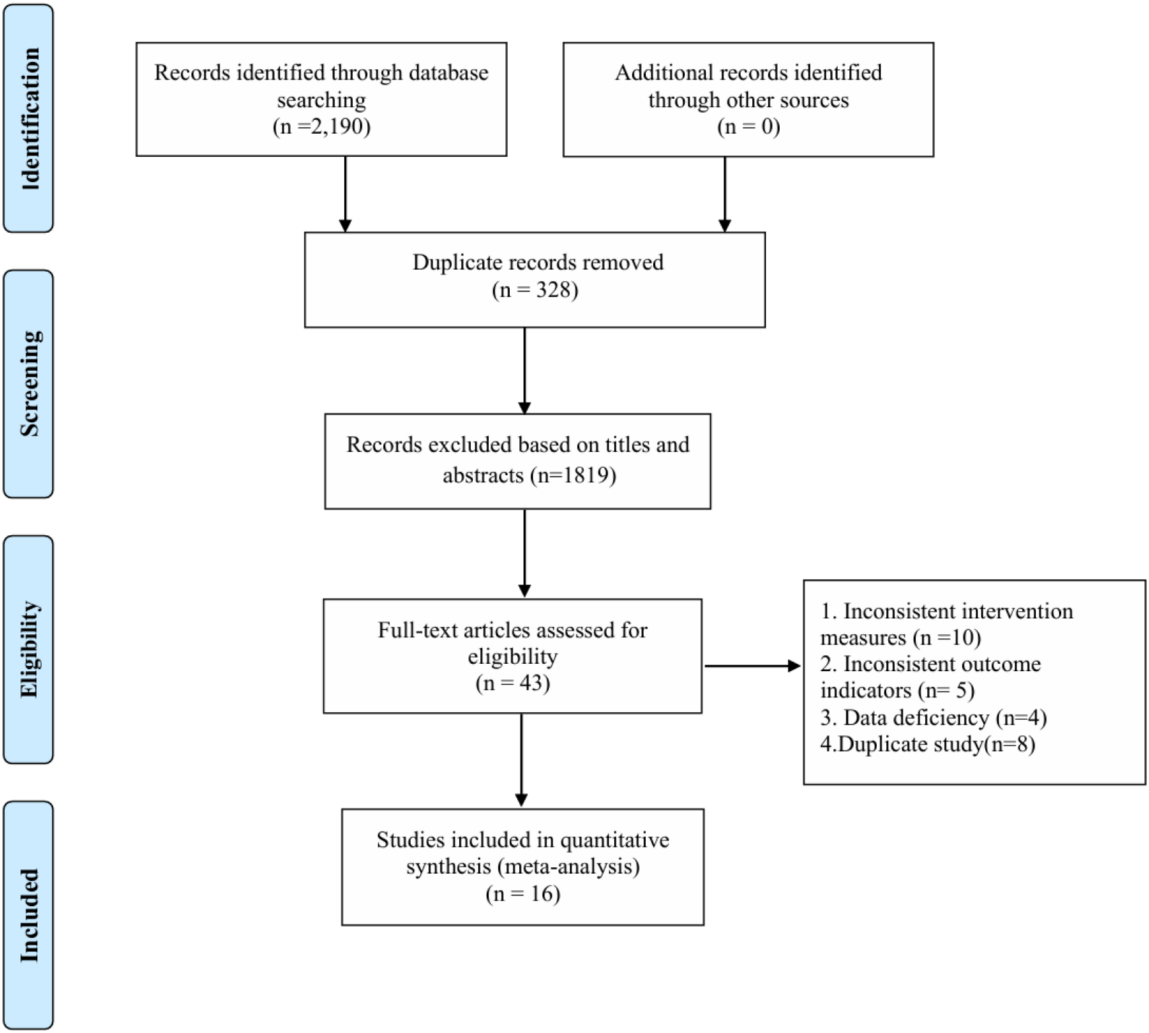
PRISM flow diagram.

After deleting duplicates, 2190 records were retrieved, 328 duplicates were removed, 1819 articles with inconsistent titles were deleted, 27 articles with inconsistent titles were removed after reading the full text, and 16 articles were finally included. The research flow chart is shown in Figure 1.

### 3.2 Basic Information Included in the Study

As shown in Table 2, the 16 included studies included 1195 teenagers addicted to mobile phones. For result measurement, 13 studies used MAPI to assess the severity of mobile phone addiction, and three studies adopted SAS‐SV. Six studies were published before 2020, and 10 studies were published after 2020.

**TABLE 2:** Detailed characteristics of the included studies.

Author	Publish Year	Country	Measure	Number	Intervention time	Intervention frequency	Outcomes
Liao [27]	2022	China	Basketball/CG	8/8	6 Weeks	2 times/week	MPAI
Wang [28]	2021	China	Basketball/CG	17/16	9 Weeks	3 times/week	SAS‐SV
Yang [29]	2022	China	AE/CG	36/36	12 Weeks	3 times/week	MPAI
Zhang [30]	2023	China	Tai chi (TC)/CG	30/30	8 Weeks	2 times/week	SAS‐SV
Zhao [31]	2020	China	OE/CG	20/16	6 Weeks	NA	MPAI
Liang [32]	2024	China	Table tennis (TT)/Bicycle/CG	20/19/18	NA	2 times/week	SAS‐SV
Wu [33]	2022	China	Table tennis (TT)/OE/CG	10/17/23	12 Weeks	NA	MPAI
Yang [34]	2020	China	Combined exercise (ce)/CG	30/30	8 Weeks	2 times/week	MPAI
Ge [35]	2015	China	OE/CG	18/18	18 Weeks	3 times/week	MPAI
Li [36]	2020	China	Combined exercise (ce)/CG	16/16	12 Weeks	NA	MPAI
Bai [37]	2022	China	Bicycle/CG	16/16	NA	NA	MPAI
Xie [38]	2019	China	Baduanjin/CG	162/152	8 Weeks	2 times/week	MPAI
Liu [39]	2019	China	Basketball/baduanjin/CG	31/31/34	12 Weeks	3 times/week	MPAI
Liu [40]	2022	China	Basketball/baduanjin/CG	31/31/34	10 Weeks	2 times/week	MPAI
Tao [41]	2021	China	Basketball/baduanjin/CG	33/33/34	10 Weeks	3 times/week	MPAI
Zhou [42]	2022	China	OE/CG	121/116	12 Weeks	Once a week	MPAI

### 3.3 Methodological Quality Assessment

**FIGURE 2:**
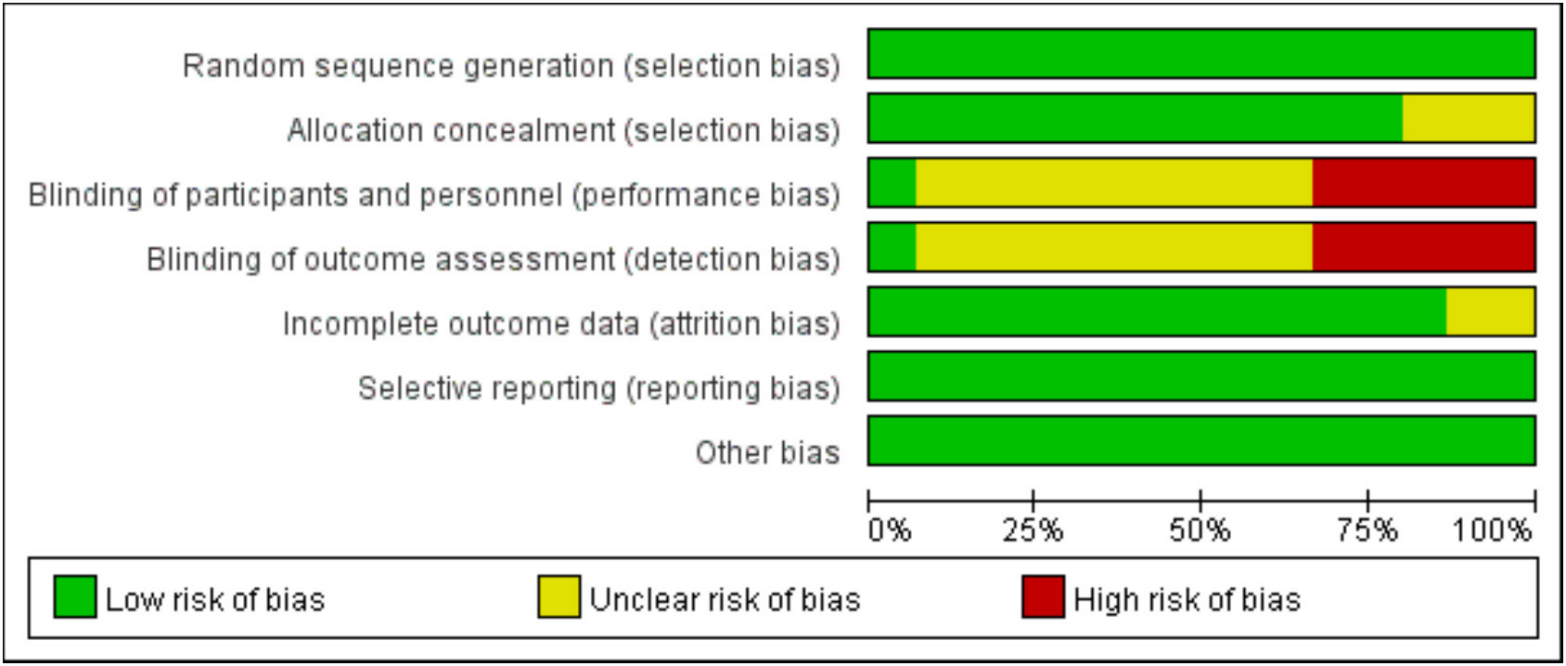
Summary of risk of bias.

The methodological quality of the included 16 articles was evaluated. Studies without high risk and those with only three or fewer studies rated as ambiguous risk were classified as low risk. There are one to five high‐risk points, but four or more are rated as high‐risk points and not explicitly classified as medium risk [43]. All other situations are high‐risk points. According to the summary of the risk of bias assessment, 12.5% (*n* = 2) of the included studies were at low risk, and 87.5% (*n* = 14) were at medium risk. The summary of the risk of bias assessment is shown in Figure 2.

### 3.4 Meta‐Analysis

**FIGURE 3:**
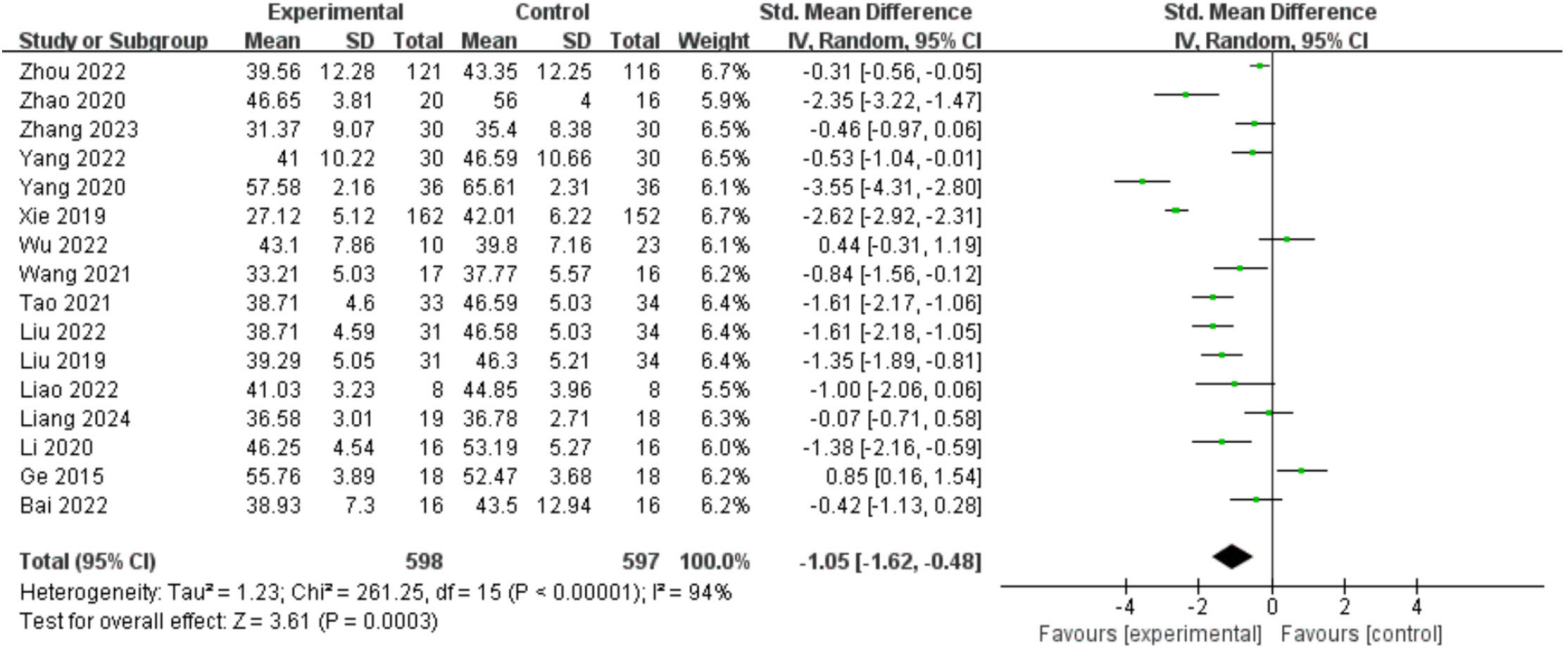
Impact of interventions on mobile phone addiction.

The effect of the exercise measures was compared with that of the control group. A meta‐analysis was conducted on 16 studies. The overall result is shown in Figure 3. Compared with the blank control group, exercise intervention can reduce the mobile phone addiction score of adolescents (SMD = −1.05, 95%CI [−1.62, −0.48], *p* < 0.001), and *I* ^2^ shows significant heterogeneity (*I* ^*2*^ = 94%, *p* < 0.001).

#### 3.4.1 Subgroup Analysis

We conducted subgroup analyses based on sample size, exercise type, outcome measures, intervention time and publication year. There were no statistically significant differences in the intervention methods, outcome measurement indicators, and publication years (*p* > 0.05). In terms of the intervention period, there was a statistically significant difference between the two subgroups (*p* < 0.05), as shown in Supplementary Table S1.

#### 3.4.2 Sensitivity Analysis

Sensitivity analysis of the included literature showed that no single study changed the overall outcome.

#### 3.4.3 Publication Bias

**FIGURE 4:**
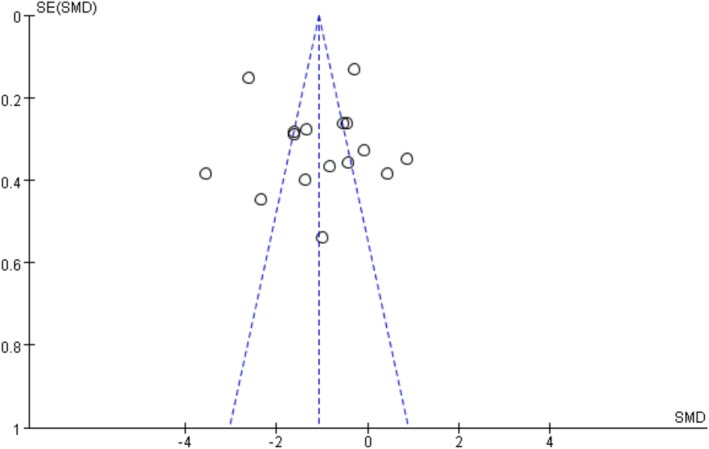
Funnel plot for the publication bias of adolescents' mobile phone addiction.

The funnel plot showed potential publication bias (Figure 4). We further conducted the test through Begg, and the result of the Begg test was *p* = 0.444 (*p* > 0.05), indicating that the probability of surface bias in the included studies was relatively low.

### 3.5 Network meta‐Analysis

To examine the differences in effects among the different interventions, network meta‐analyses were further performed.

#### 3.5.1 Network Diagram

**FIGURE 5:**
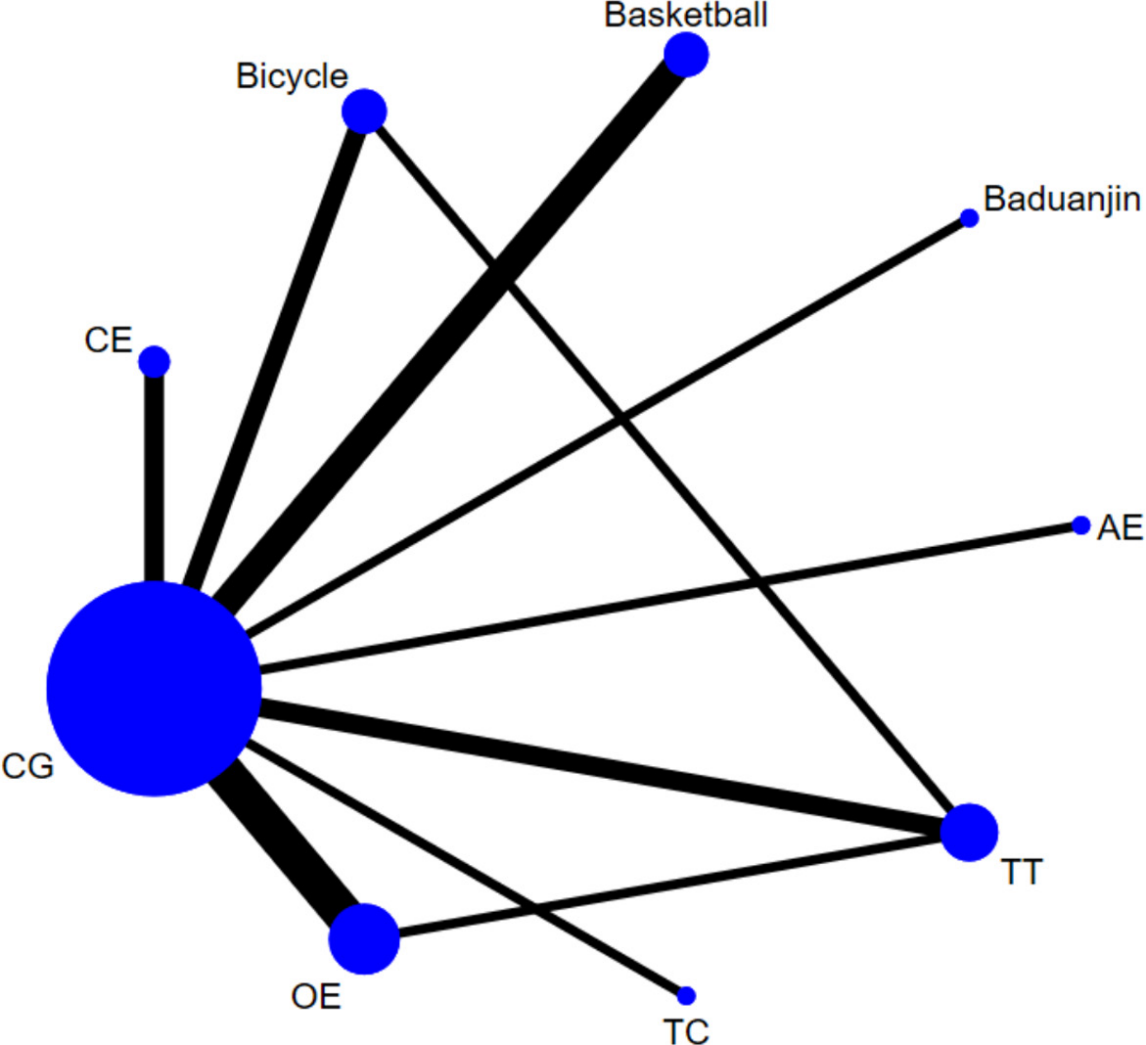
Network diagram of Mobile phone addiction. Aerobic exercise (AE), tai chi (TC), table tennis (TT), combined exercise (ce), control group (CG), other exercises (OE).

As shown in Figure 5, the dots in the figure represent the number of subjects in each group; the larger the dots are, the larger the sample size of the subjects. The lines connecting the dots represent the number of original studies directly compared in pairs; the thicker the lines are, the more original studies there are.

#### 3.5.2 Inconsistency of the Network

The inconsistent model was used for the test. The result (*p* = 0.0013) indicated that the inconsistent model was significant. Then, the sources of inconsistency needed to be analysed and eliminated. The local inconsistency test used the point division method to find the sources of inconsistency. It was found that the sources of inconsistency were the studies of Tao [41] and Liu [40]. After the study was deleted, tests for inconsistency and local inconsistency were conducted again, and neither was significant.

#### 3.5.3 Contribution Plot

The contributions of direct and indirect comparisons to network meta‐analysis and the number of studies of each direct comparison are shown in Supplementary Figure S1.

#### 3.5.4 Results of Network Meta‐Analysis

**FIGURE 6:**
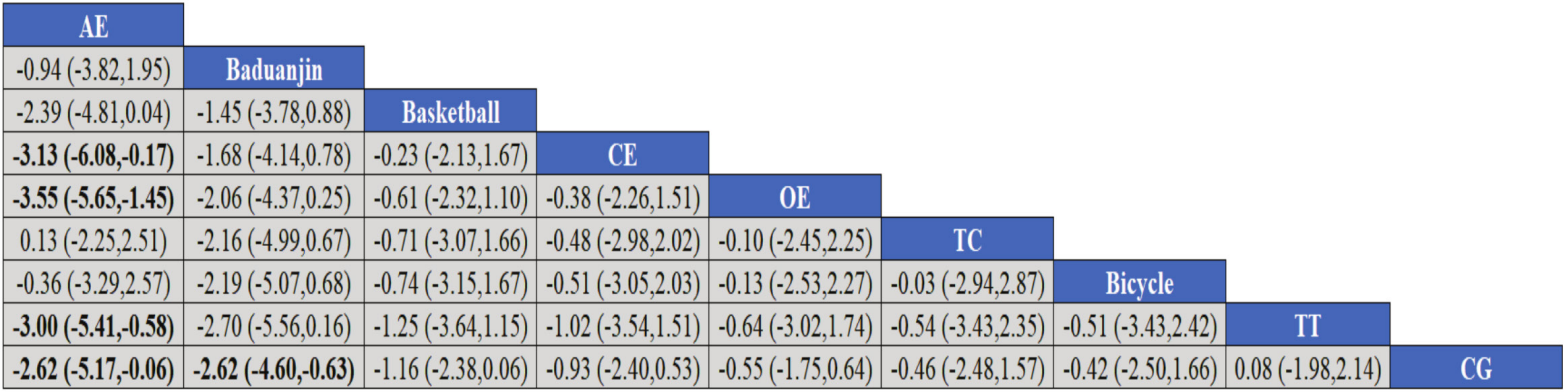
Forest plot for network meta‐analysis.

NMA showed that AE was significantly superior to basketball (SMD ‐3.13, 95% ‐6.08 to −0.17), OE (SMD ‐3.55, 95% ‐5.64 to −1.45), TT (SMD ‐3.00, 95% ‐5.41 to −0.58), CG (SMD ‐2.62, 95% ‐5.17 to −0.06). As shown in Figure 6, the forest plots of the comparisons that meet the conditions are shown in Supplementary Figure S2.

#### 3.5.5 Intervention Effect Ranking

The SUCRA probability of each intervention in the network is shown in Supplementary Figure S3. The SUCRA value (Table 3) is the probability that each intervention is among the best of those in the network, with larger values representing higher‐ranking probabilities.

**TABLE 3:** The SUCRA values of the interventions.

Treatment	SUCRA
A = Aerobic exercise (AE)	95.6
B=Baduanjin	85.8
C = Basketball	59.4
D = Bicycle	36.5
E = Combined exercise (CE)	52.4
F = Control group (CG)	19.1
G = Other exercises (OE)	40.3
H = Tai chi (TC)	36.9
I = Table tennis (TT)	23.9

#### 3.5.6 Risk of Bias Across Studies

**FIGURE 7:**
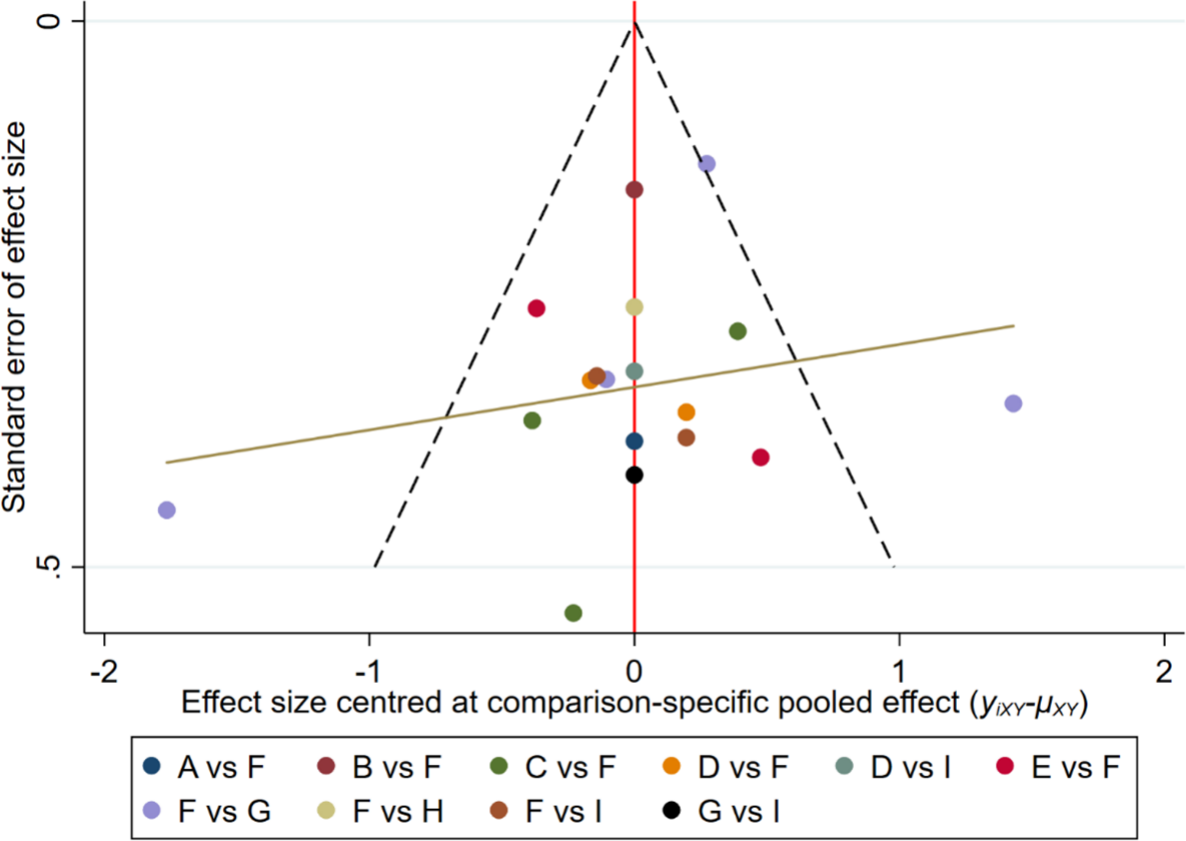
Comparison‐adjusted funnel plot of adolescent mobile phone addiction scores. A = aerobic exercise (AE), B = baduanjin, C = basketball, D = bicycle, E = combined exercise (CE), F = control group (CG), G = other exercises (OE), H = tai chi (TC), I = table tennis (TT).

The publication bias was illustrated by funnel plots (Figure 7). According to the network meta‐analysis, the funnel plot showed slight asymmetry.

## 4 Discussion

This study included randomised controlled studies from seven databases. To evaluate the effect of the intervention and treatment control, a traditional meta‐analysis was conducted on 14 studies. Furthermore, a network meta‐analysis was conducted on 9 different exercise intervention measures in 14 studies to analyse the direct and indirect comparisons among different exercise measures.

### 4.1 Meta‐Analysis

We conducted a meta‐analysis of 14 randomised controlled trials to assess the impact of exercise intervention on mobile phone addiction. The results showed that compared with the untreated control group, exercise intervention could effectively reduce the mobile phone addiction score. Total dose effect (SMD = −1.05, 95% CI [−1.62, −0.48], *p* < 0.001). Physical exercise is a physical activity that takes physical movement as its content and means, aims at improving physical health, and has a certain intensity, frequency, and duration [44]. The Use and Satisfaction Theory (UGT) holds that individuals use new media to meet their various needs, which is widely adopted in media communication research [45, 46]. According to this theory, the smartphone is a ‘ritualised’ medium used to meet people's needs for transmitting time, relaxation and entertainment, and it is easy to form habits [47]. The psychological needs of individuals, such as socialising and entertainment, are met to varying degrees with the use of smartphones. This sense of satisfaction prompts individuals to become dependent on network devices such as mobile phones [48], leading to mobile phone addiction [49]. The functions of physical exercise, such as leisure and entertainment, cultivating one's sentiments and interpersonal communication [50], can also meet the psychological needs of college students. However, due to the significant heterogeneity among the studies, the research results can only be considered preliminary.

### 4.2 Network Meta‐Analysis

On this basis, the network meta‐analysis was carried out further to analyse the matched intervention effect of each exercise intervention, and the intervention measures were ranked.

Our research results show that AE can effectively reduce the score of mobile phone addiction. AE is a kind of physical activity that integrates dance, music, and gymnastics and achieves the purposes of fitness and mental health through body weight or equipment and other forms [51]. It has a wide range of applications, is not restricted by location, and has good ornamental value. In addition, AE accompanied by music can stimulate the brain to produce endorphin neurotransmitters, which can relieve negative emotions such as anxiety and depression among female college students, maintain a good mood, release psychological pressure, divert attention from mobile phones during spare time and enrich extracurricular life [52]. This can effectively reduce the situation where female college students fall into a state of mobile phone addiction due to seeking emotional satisfaction and stress relief. Through group arrangement and cooperation in sports, students can improve their interpersonal communication and mutual communication skills, create a good interpersonal communication atmosphere, make sports a good channel to make like‐minded friends, love sports, gain more social activities and happiness in sports, reduce anxiety and depression, and lower their attention to mobile network social interaction. Being willing to spend more time participating in AE reduces the time spent on mobile phones and decreases the degree of mobile phone addiction.

### 4.3 Strengths and Limitations

It is crucial to identify and explain some advantages and limitations of this study. Mobile phones have become an important medium of information, featuring personalisation and unparalleled accessibility [53]. Especially for teenagers, mobile phones have become indispensable tools [53]. Excessive use of mobile phones can make teenagers addicted to them, thereby leading to a series of adverse consequences. Our research results confirm the effectiveness of exercise intervention in improving mobile phone addiction and further explore which kind of exercise has the best effect on improving mobile phone addiction by using network element analysis. Future research will provide suggestions for the selection of exercise interventions. First, we conducted a comprehensive and systematic search of the published literature to reduce bias and identify potentially related studies. Secondly, this study adopted the retrieval method to search seven databases, and the retrieved literature was analysed. We conducted a strict literature screening. Finally, we included 14 studies. Many of the studies we included were published in Chinese, and the results should be inferred with caution. Finally, exercise intervention measures are ranked based on the average SUCRA score. This does not necessarily mean that the intervention measures with a higher ranking are statistically significantly superior to those with a lower ranking. Therefore, the research results should be interpreted with caution. Our research confirms that exercise has a very good effect on improving mobile phone addiction. However, which form and intensity of exercise best improves mobile phone addiction remains to be further explored. We suggest that in future research, attention should be paid to the mobile phone addiction behaviours of different groups of people, the impact of an exercise intervention on the addiction problems of other groups in society should be understood from multiple dimensions, and the health and quality of life of the target population should be improved in a targeted manner. In our study, only 12.5% of the included RCTs were rated as having a low risk of bias, with the majority belonging to the medium‐risk category. Among the studies we included, there are still a few that did not specify the specific randomisation methods. There is a lack of clear coverage of the hidden and blind parts. The ambiguity of the methodological quality designed in these studies may reduce the strength of the evidence quality and further affect the robustness of our results. We suggest that in future research, the quality of jurisprudence should be further controlled to minimise its impact on the results. Therefore, in the future, more rigorous, comprehensive, and high‐quality randomised controlled trials with different cultural backgrounds should be conducted to provide a reliable theoretical basis for research updates in this field. In addition, neuroscience tools such as electroencephalogram (EEG) and transcranial magnetic stimulation can be considered to study the impact of exercise on mobile phone addiction to better address the underlying mechanisms.

## 5 Conclusion

According to our research results, all exercise interventions have a significant impact on improving mobile phone addiction. Based on the network meta‐analysis results, aerobic exercise may be the best intervention measure. We will continue incorporating additional exercise intervention measures to provide evidence for researchers to select the best exercise measures.

## Author Contributions

**Ying Li:** conceptualisation, methodology, data curation, writing – original draft, writing – review and editing. **Jianhua Zhang:** methodology, data curation, writing – original draft, writing – review and editing. **Zhaojun Luo:** conceptualisation, data curation, writing – original draft. **Dan Tao:** conceptualisation, data curation, writing – original draft. **Lizhong Wen:** methodology, data curation, writing – original draft.

## Conflicts of Interest

The authors declare no conflicts of interest.

## Supporting information


**TABLE S1:** Subgroup analysis to assess the effect of interventions on adolescents' intervention addiction.
**FIGURE S1:** Contribution plot.
**FIGURE S2:** Results of network meta‐analysis.
**FIGURE S3:** SUCRA graph of effectiveness among interventions.

## Data Availability

Data sharing is not applicable to this article as no datasets were generated or analysed during the current study.
